# Complete Mitochondrial Genome of *Haemulon plumierii* (Lacepède, 1801) Supports Its Use as a Sentinel Reef Fish

**DOI:** 10.3390/genes17050585

**Published:** 2026-05-20

**Authors:** Mayra Alejandra Cañizares-Martínez, Jesús Alejandro Zamora-Briseño, Rafael F. Rivera-Bustamante, Rossanna Rodríguez-Canul

**Affiliations:** 1 Departamento de Recursos del Mar, Centro de Investigación y de Estudios Avanzados del Instituto Politécnico Nacional Cinvestav-IPN, Unidad Mérida, Carretera Antigua a Progreso Km. 6, Mérida 97310, Yucatán, Mexico; mayra.canizares@cinvestav.mx; 2 Red de Estudios Moleculares Avanzados, Campus III, Instituto de Ecología A. C., INECOL, Carretera Antigua a Coatepec 351, Xalapa 91073, Veracruz, Mexico; alejandro.zamora@inecol.mx

**Keywords:** mitochondrial genome, chac-chic, *Haemulon plumierii*, oxidative phosphorylation, sentinel species

## Abstract

**Background**: Mitochondrial genomes provide valuable information on evolutionary relationships among organisms and on the selective pressures acting on energy metabolism, increasing their relevance in ecological and environmental genomics studies. *Haemulon plumierii* is a reef-associated fish distributed throughout the Gulf of Mexico and Caribbean Sea and has been proposed as a bioindicator species within the Mesoamerican Reef System. **Methods**: In this study, we present a high-quality mitochondrial genome of *H. plumierii* from the southeastern coast of Mexico generated using PacBio HiFi long-read sequencing. **Results**: The circular mitogenome is 16,823 bp long and contains the complete set of 37 canonical mitochondrial genes, including 13 protein-coding genes, 22 tRNAs, two rRNAs, and one control region (D-loop). The gene order, strand orientation, and tRNA secondary structures were consistent with the conserved vertebrate mitochondrial architecture. Comparative analyses with closely related haemulid species revealed conserved nucleotide composition patterns, negative GC skew values, strong AT enrichment within the D-loop, and highly conserved mitochondrial synteny. Phylogenetic reconstruction based on complete mitochondrial genomes placed *H. plumierii* firmly within the *Haemulon* clade. Selective pressure analyses revealed pervasive purifying selection acting on mitochondrial protein-coding genes, supported by low dN/dS ratios, high amino acid identity, constrained nucleotide diversity in cytochrome oxidase genes, and conserved codon usage patterns shaped primarily by AT-driven mutational bias. Pairwise genetic distance analyses further supported moderate interspecific divergence within Caribbean *Haemulon* species. **Conclusions**: Overall, the mitogenomic resource generated here provides an important evolutionary and functional framework for future phylogenetic, ecological, and environmental genomics studies in Caribbean reef fishes.

## 1. Introduction

Mitochondria are essential organelles responsible for cellular energy production through oxidative phosphorylation and play a central role in the regulation of reactive oxygen species (ROS) homeostasis, programmed cell death, innate immune signaling, and cellular stress responses [1,2]. Because mitochondrial function is tightly linked to cellular metabolism, perturbations affecting mitochondrial pathways can have profound physiological consequences at the organism level.

Mitochondrial DNA (mtDNA) encodes key subunits of the respiratory chain complexes, that maintain mitochondrial integrity and bioenergetic performance [3]. In contrast to nuclear DNA, mtDNA lacks protective histones and exhibits limited DNA repair capacity, rendering it particularly susceptible to damage induced by oxidative stress and exposure to environmental contaminants [4,5,6]. Accumulation of mtDNA damage has been associated with mitochondrial dysfunction and it has been also implicated in aging, metabolic disorders, and stress-related pathologies [7].

Fish mitochondrial genomes are organized as a closed, circular, double-stranded extranuclear molecules composed of a heavy strand (H) and a light strand (L) [8]. They are compact and conserved, with lengths varying from 15 to 18 kb. They possess a standardized genetic composition that includes 13 protein-coding genes associated with oxidative phosphorylation, 22 transfer RNA genes, two ribosomal RNA genes, and a non-coding control region (D-loop) [9,10]. This conserved organization, along with the functional relevance of mitochondrial genes, has made complete mitogenomes valuable resources for evolutionary, comparative, and environmental genomics. Indeed, previous studies have shown that complete mitochondrial genomes provide robust resolution of phylogenetic relationships, facilitate comparative analyses of genome organization, and serve as reliable reference genomes in various teleost lineages, as well as useful biomarkers of xenobiotic exposure [11,12,13].

Beyond their usefulness in evolutionary studies, mitochondrial genomes have gained increasing relevance in environmental and ecotoxicological research. Genes encoding components of the electron transport chain, such as *nad*, *cox*, and *cob*, are located near ROS generation sites and are, therefore, particularly sensitive to physiological alterations associated with chemical stressors [14,15]. Alterations in mitochondrial gene expression, sequence integrity, or genome organization have been widely linked to metabolic disruption, oxidative stress, and reduced performance in fish exposed to environmental pollutants [6,16]. Consequently, high-quality mitochondrial genomic resources provide the molecular basis to investigate the sublethal effects of environmental pollution and for developing mitochondria-based biomarkers in aquatic organisms.

The Gulf of Mexico (GoM) is one of the world’s most industrialized marine regions and has experienced chronic hydrocarbon pollution associated with offshore oil extraction, hydrocarbons shipping, coastal urbanization, and episodic oil and gasoline spills [17,18]. Petroleum-derived compounds, including polycyclic aromatic hydrocarbons (PAHs), persist in marine sediments and coastal ecosystems, creating long-term exposure scenarios for resident species [19]. Toxicological studies in fish have shown that exposure to hydrocarbons and hypoxic conditions induces important alterations in mitochondrial bioenergetic and redox pathways [20,21]. Therefore, reliable mitochondrial genomic references are essential for future transcriptomic, mitogenomic, and ecotoxicological studies aimed at evaluating the molecular responses of marine organisms to environmental contamination in the Gulf of Mexico and Caribbean region.

*H. plumierii* (Lacepède, 1801), commonly known as the white grunt (locally known as “chac-chic”) in the Mexican Caribbean, is a reef-associated fish belonging to the family *Haemulidae*, and it is widely distributed throughout the western Atlantic, including the GoM and the Caribbean Sea [22]. This species inhabits coral reefs, rocky substrates, and soft-bottom habitats at shallow to moderate depths and plays an important trophic role as an intermediary between benthic invertebrates and apex predators [23,24]. Due to its ecological relevance, wide geographic distribution, site fidelity, and local abundance, *H. plumierii* has been selected as a bioindicator species within reef monitoring programs, including the Mesoamerican Reef System [25]. Experimental evidence also indicates that this species has behavioral and physiological responses to hypoxia and other environmental stressors, which further supports its sensitivity to xenobiotic agents [26,27].

Despite its ecological importance and its potential use as a sentinel species, the genomic resources of *H. plumierii* remain limited. Although a mitochondrial genome for this species has been previously deposited in GenBank (OP056946.1), the present study provides the first mitogenome assembled using PacBio HiFi long-read sequencing technology, allowing improved assembly confidence, enhanced structural validation, and better characterization of repetitive and control-region features compared with conventional short-read assemblies [28].

The objective of this study was to describe the complete mitochondrial genome of *H. plumierii*, generated using high-fidelity long-read sequencing, and to perform comparative analyses within representative haemulid taxa. In addition to structural annotation and phylogenetic reconstruction, we evaluated selective pressure patterns, codon usage bias, nucleotide diversity, sequence divergence, and control-region organization across haemulid mitogenomes to better understand patterns of mitochondrial conservation and evolution within the group [29]. This mitogenome constitutes a valuable genomic resource for comparative evolutionary studies and provides a robust molecular framework supporting future environmental genomics, ecotoxicological, and biomonitoring applications focused on mitochondrial responses to environmental stress in tropical marine ecosystems.

## 2. Materials and Methods

### 2.1. Ethical Statement

All live animal protocols described below were approved by the Institutional Animal Care and Use Committee of Cinvestav (CICUAL-Cinvestav) (Reference number: 0126-15) guidelines, following the Mexican Official Standard NOM-0612-ZOO-1999.

### 2.2. Sample Collection, DNA Extraction, and Sequencing

Five juvenile specimens of *H. plumierii* were collected near the coast of Celestún, Yucatán, Mexico, in the southern GoM (20°49′40.00″ N, 90°25′22.88″ W). Fish were anesthetized with Tricaine Methane Sulfate (MS222), and gill tissues were dissected immediately after collection and rinsed with sterile 1× phosphate-buffered saline (PBS) to remove residual blood and surface contaminants. Tissues were preserved in liquid nitrogen and transported to the Laboratory of Biotechnology and Molecular Toxicology at the Center for Research and Advanced Studies of the National Polytechnic Institute (Cinvestav), Mérida, Mexico, where they were stored at −80 °C until DNA extraction.

Total genomic DNA was extracted from each organism using the DNeasy Blood & Tissue Kit (QIAGEN, Hilden, Germany). DNA concentration and purity were evaluated using NanoDrop spectrophotometry (Thermo Fisher Scientific, Waltham, MA, USA) and Qubit 3.0 fluorometry (Invitrogen, Carlsbad, CA, USA). DNA integrity was assessed by electrophoresis on 1.5% agarose gels stained with GelRed and visualized under UV illumination.

The highest-quality genomic DNA sample from one of the five collected organisms was selected for long-read whole-genome sequencing. Mitochondrial genome assembly was intentionally performed using a single individual to avoid the incorporation of multiple mitochondrial haplotypes that could interfere with de novo assembly accuracy, consistent with standard practices in mitogenome assembly studies using long-read sequencing technologies. The selected DNA sample was sent to Macrogen Inc. (Seoul, Republic of Korea) for library preparation using the PacBio HiFi protocol. Sequencing was conducted on the PacBio Revio platform using SMRT Cells, generating circular consensus (HiFi) reads.

### 2.3. Mitochondrial Genome Assembly and Annotation

The mtDNA of *H. plumierii* was assembled using high-fidelity long reads generated by PacBio sequencing. Mitochondrial reads were identified and assembled de novo using MitoHiFi v3.2.2 [30], a pipeline specifically designed for accurate reconstruction of circular mitochondrial genomes from HiFi data. The previously published mitochondrial genome of *H. plumierii*, available in the NCBI database (accession OP056946.1), was used exclusively to guide read identification and assembly validation without enforcing a reference-based assembly strategy.

To ensure the assembly of a true mitochondrial genome and avoid the inclusion of nuclear mitochondrial DNA (NUMTs), we employed the MitoHiFi pipeline, which filters high-fidelity reads based on their homology to reference mitogenomes. The circularity of the resulting contig was confirmed through an automated circularization check that identified consistent overlapping sequences at the contig ends.

Structural and functional annotation of the assembled mitogenome was initially performed using MitoFinder v1.4.1 [31] and independently verified using the MITOS2 v2.1.10 [32] web server under the vertebrate mitochondrial genetic code. The annotation process identified protein-coding genes, ribosomal RNA genes, transfer RNA genes, and non-coding regions. Transfer RNA secondary structures were additionally evaluated using tRNAscan-SE [33] to confirm canonical mitochondrial tRNA folding patterns.

A circular graphical representation of the annotated mitochondrial genome, including gene order, transcriptional orientation, and functional categories, was generated using MitoFish v4.09 [34]. Base composition analyses, including nucleotide frequencies (A, T, G, and C), A+T and G+C contents, and AT and GC skew values, were calculated for complete mitochondrial genomes and control regions to evaluate compositional biases and strand asymmetry among Haemulid mitogenomes. Comparative genomic analyses were performed using complete mitochondrial genomes retrieved from GenBank.

Tandem repeat content across the complete mitochondrial genomes was evaluated using Tandem Repeat Finder v4.09 [35] to assess repetitive elements in coding and non-coding regions, including the mitochondrial control region (D-loop).

### 2.4. Assembly Quality and Validation

The quality and accuracy of the assembly were assessed using multiple independent and complementary validation strategies. Using minimap2 v2.24 [36], PacBio HiFi reads were mapped to the assembled mitochondrial genome. This enabled evaluation of read support, coverage uniformity, and assembly continuity. For a further independent validation of the assembly, Illumina short-read sequencing data from a previously published *H. plumierii* genome available at NCBI (FW: SRR30553176_1.fastq; RV: SRR30553176_2.fastq) were aligned with the assembled mitochondrial genome. These Illumina reads originated from an independent specimen collected from the US coast and were not used during assembly, thereby providing an external source for sequence validation.

Annotation consistency and gene boundary accuracy were additionally evaluated through comparison between MitoFinder and MITOS2 outputs. The final annotated mitogenome was formatted and validated for GenBank submission using tbl2asn.

Predicted secondary structures of the 22 mitochondrial tRNA genes were inferred using ViennaRNA v2.7.0 [37], allowing evaluation of canonical cloverleaf conformations. Final organization and visualization of tRNA secondary structure figures were prepared using Inkscape v1.2 (2022) [38].

### 2.5. Phylogenetic Analysis and Comparative Sequence Similarity

To assess the phylogenetic assignment of *H. plumierii* and the consistency of the assembled mitogenome with established taxonomic relationships, the complete mitochondrial genome sequences from 17 closely related haemulid species were retrieved from the NCBI database. *Danio rerio* was included as an outgroup.

Complete mitochondrial genome sequences were aligned using MAFFT v7 [39] with default parameters optimized for long and highly similar sequences. Phylogenetic inference was performed under the maximum likelihood framework using IQ-TREE 3 [40]. Model selection was performed with ModelFinder [41], and the TPM2u+F+I+G4 substitution model was selected as the best-fit model under the Bayesian Information Criterion (BIC). Branch support was assessed using 1000 ultrafast bootstrap replicates [42]. The resulting phylogenetic tree was visualized using iTOL v5 [43].

To complement phylogenetic inference, sequence similarity among mitochondrial genomes was assessed using BLAST v2.9 [44] (pairwise comparisons). Pairwise alignments were conducted between the assembled *H. plumierii* mitochondrial genome and complete mitogenomes of closely related haemulid taxa retrieved from NCBI (Supplementary Table S1) to assess sequence conservation, alignment coverage, and overall genomic similarity.

Based on BLAST similarity results and phylogenetic proximity, a subset of seven closely related haemulid species was selected for comparative genomic analyses. Gene order conservation and mitochondrial synteny among these taxa were visualized using Easyfig v2.2.5 [45], which generates linear comparative maps based on annotated GenBank files. Default parameters were applied, and homologous regions were identified using BLASTn v2.12 [46] within Easyfig to illustrate conserved gene blocks, transcriptional orientation, and structural organization across mitogenomes.

### 2.6. Selection Pressure Analysis of Mitochondrial Protein-Coding Genes

To evaluate selective constraints acting on mitochondrial protein-coding genes, analyses of nonsynonymous (dN) and synonymous (dS) substitution rates were performed. Protein-coding gene sequences were extracted from annotated mitochondrial genomes of seven *Haemulidae* species, corresponding to the same taxa selected based on the initial BLAST similarity analysis and phylogenetic clustering (see above). These species represent closely related Caribbean lineages and were retained to ensure comparability and minimize phylogenetic noise.

In the subsequent analyses, only complete protein-coding genes with consistent lengths and reliable annotations were included. Nucleotide sequences were translated using the vertebrate mitochondrial genetic code. Amino acid alignments were generated independently for each gene using MAFFT v7 [47], using default parameters. These amino acid alignments were then used to construct codon-based nucleotide alignments, preserving the original reading frames and avoiding the introduction of frameshifts.

Estimation of dN, dS, and dN/dS (ω) ratios was performed using the CODEML program implemented in the PAML v4.10 package [48]. A one-ratio model was applied, assuming a single ω value across all branches and sites for each gene. Analyses were conducted using gene-specific phylogenetic trees derived from the same seven species included in each codon alignment. Model parameters, including the transition/transversion rate ratio (κ) and ω, were estimated directly from the data.

To assess differences in selective pressure across mitochondrial functional groups, ω (dN/dS) values were compared among gene complexes using a the non-parametric Kruskal–Wallis test, implemented in R v4.5.1 [49], selected due to the limited sample size and distribution-independent nature of the data.

### 2.7. Codon Usage Bias, Nucleotide Diversity, and Genetic Distance Analyses

Relative synonymous codon usage (RSCU) values were calculated from the concatenated alignment of the 13 mitochondrial protein-coding genes (PCGs). Prior to RSCU estimation, alignment gaps were removed and sequences were trimmed to preserve complete codon frames. Stop codons corresponding to the vertebrate mitochondrial genetic code (TAA, TAG, AGA, and AGG) were excluded from heatmap visualization analyses. RSCU values were calculated in R using the package seqinr [50], and heatmap visualizations were generated using ggplot2 [51].

Nucleotide diversity across the concatenated alignment of the 13 mitochondrial protein-coding genes was evaluated using a sliding-window analysis implemented in R. Analyses were performed using a window size of 200 bp and a step size of 20 bp. Pairwise nucleotide differences were calculated excluding gaps and ambiguous sites.

Pairwise genetic distances among taxa were estimated using the Kimura 2-parameter (K2P) model [52] implemented in the R package ape [53] based on the concatenated alignment of the 13 mitochondrial protein-coding genes. Heatmap visualizations of K2P genetic distances were generated using ggplot2 [51].

### 2.8. D-Loop Characterization

The mitochondrial control region (D-loop) was identified as the largest intergenic non-coding region in each mitogenome. For *H. plumierii* and comparative taxa, D-loop length and nucleotide composition were calculated, including base frequencies (A, T, G, C), AT and GC content, and AT and GC skew values. These analyses were performed using custom Python v3.9 [54] scripts based on Biopython v1.85 [55].

## 3. Results

### 3.1. Sample Collection, DNA Extraction, and Sequencing

High-molecular-weight genomic DNA extracted from *H. plumierii* met all quality control requirements for PacBio HiFi library preparation. The final sequencing library exhibited a concentration of 31.8 ng/µL with a fragment size distribution optimized within the 10–50 kb range. Sequencing on the PacBio Revio platform generated 591,345 HiFi reads (~6.7 Gb) with a mean length and N50 of 11.3 kb and an average quality score of Q30.

### 3.2. Mitochondrial Genome Assembly and Annotation

**Figure 1:**
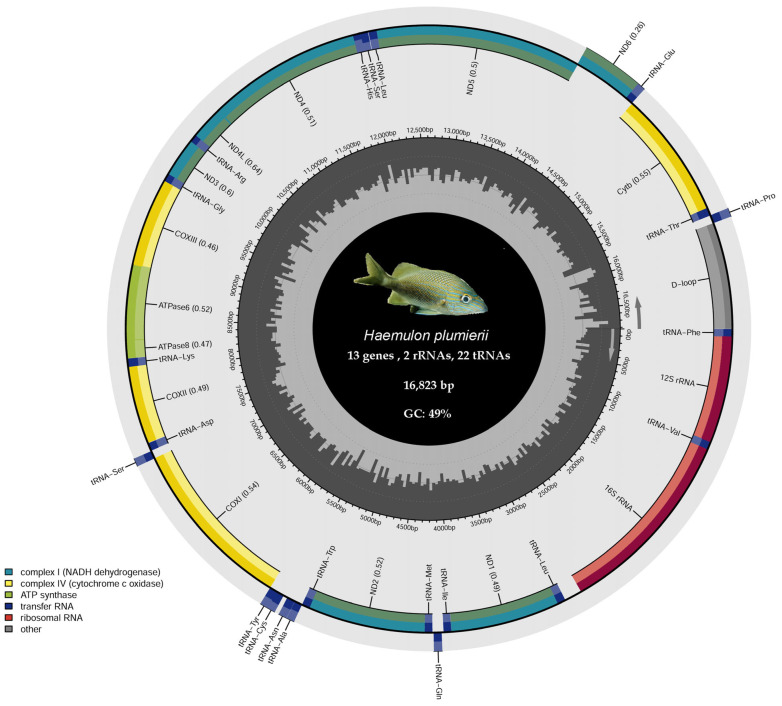
Circular representation of the annotated mitochondrial genome of *H. plumierii*. Protein-coding genes (PCGs), transfer RNA (tRNA) genes, ribosomal RNA (rRNA) genes, and the control region (D-loop) are shown according to their relative genomic positions and transcriptional orientation. Genes encoded on the heavy (H) and light (L) strands are indicated by the direction of the arrows. Protein-coding genes involved in oxidative phosphorylation are color-coded according to their functional complexes. The inner histogram represents variation in GC content along the mitochondrial genome calculated using a sliding-window approach.

The complete mitogenome of *H. plumierii* is a circular molecule of 16,823 bp (GC content: 48.78%; NCBI: PZ295672). It follows the typical vertebrate organization, containing 37 genes: 13 protein-coding genes (PCGs), 22 transfer RNAs (tRNAs), two ribosomal RNAs (*12S* and *16S*), and a non-coding control region (Figure 1). Most genes are encoded on the heavy strand (H-strand), except for *nad6* and eight tRNAs (*tRNA-Gln*, *-Ala*, *-Asn*, *-Cys*, *-Tyr*, *-Ser* (*UCN*), *-Glu*, and *-Pro*), which are located on the light strand (L-strand) (Table 1).

The nucleotide composition shows a slight AT preference (51.22%). Among the 13 PCGs, the majority use ATG as the start codon, although *atp6* and *cox1* exhibit alternative initiation. Notably, seven PCGs (*nad2*, *cox2*, *atp6*, *cox3*, *nad3*, *nad4*, and *cob*) terminate with incomplete stop codons (T or TA), which are common in teleosts and presumed to be completed via post-transcriptional polyadenylation. The ribosomal genes, *rrnS* (949 bp) and *rrnL* (1682 bp), are positioned between *tRNA-Phe* and *tRNA-Leu* (*UUR*), separated by *tRNA-Val*.

**Figure 2:**
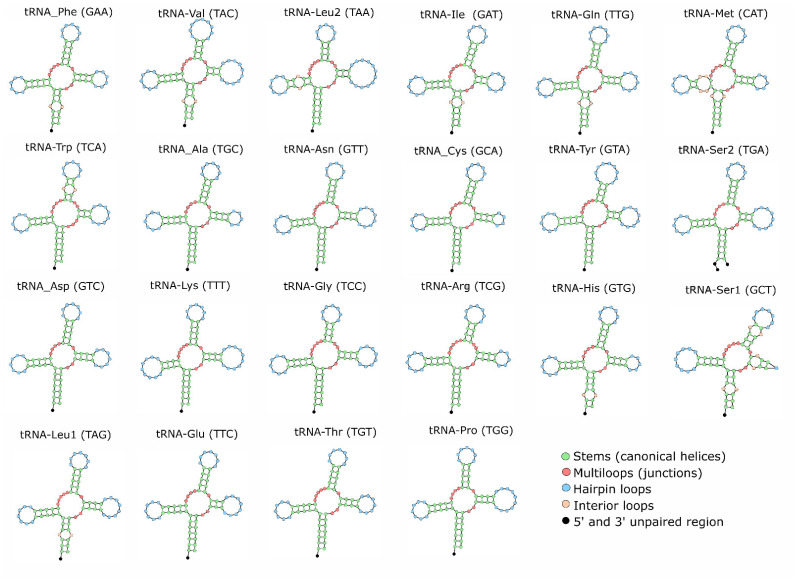
Putative secondary structure of 22 tRNA identified in *H. plumierii* mitogenome. tRNAs are shown in order of occurrence in the mitogenome starting from tRNA-Phe. Anticodons are in parentheses after the name of each tRNA.

The 22 tRNA genes range in length from 66 to 75 bp (Table 2). No evidence of gene duplication or rearrangement was found, indicating high structural conservation. Based on the predicted secondary structures, most tRNAs display a canonical cloverleaf conformation (Figure 2). However, *tRNA-Ser* (*AGN*) lacks a functional DHU arm, a characteristic feature observed in vertebrate mitochondrial genomes.

The mitochondrial genome of *H. plumierii* showed a nucleotide composition slightly biased toward AT (A+T = 51.22%), with base frequencies of 26.04% A, 25.19% T, 17.55% G, and 31.23% C (Table 3). This pattern falls within the range observed in the genus *Haemulon*, whose species showed similar A+T contents (50.43–51.37%) and GC values close to 49%, indicating strong nucleotide compositional conservation among them. In contrast, the outgroup *D. rerio* exhibited a notably higher A+T content (60.07%) and lower GC content (39.93%), consistent with its more distant phylogenetic position and lineage-specific mitochondrial genomic characteristics.

Analysis of nucleotide skews revealed conserved patterns of compositional asymmetry among haemulid mitogenomes. The mitochondrial genome of *H. plumierii* exhibited a weakly positive AT skew (0.017), indicating a slight excess of adenine over thymine, and a strongly negative GC skew (−0.280), reflecting an excess of cytosine relative to guanine. Comparable skew values were consistently observed across other *Haemulon* species, with AT skew values generally close to zero and GC skew values ranging from −0.27 to −0.29. This shared compositional bias suggests that *Haemulon* mitogenomes are shaped by similar strand-specific mutational pressures and conserved mitochondrial replication mechanisms.

Haemulid taxa outside the genus *Haemulon*, including *Anisotremus surinamensis*, *Conodon nobilis*, *Pomadasys kaakan*, and *Orthopristis chrysoptera*, showed similar overall compositional trends, although slightly greater variability was observed in some lineages. In contrast, *Lutjanus argentimaculatus* displayed a distinct nucleotide composition characterized by elevated guanine content, differentiating it from haemulid mitogenomes and supporting its phylogenetic separation from the family *Haemulidae*.

### 3.3. Assembly Quality and Validation

Independent validation analyses confirmed the structural accuracy and completeness of the *H. plumierii* mitogenome. Circularization was computationally verified using this check module in MitoHiFi v3.2.2. The circularization analysis identified a significant sequence overlap between the ends of the assembled contig (ptg000001l), ensuring the recovery of a complete and intact circular molecule of 16,823 bp. Proper genome orientation and structural integrity were further supported by the canonical positioning of the *tRNA-Phe* gene at the start of the sequence and the successful reconstruction of the control region (D-loop).

The assembly showed robust read support and overall high sequence coverage. Mapping of PacBio HiFi reads to the consensus sequence resulted in a mean depth of 54.4×, with ≥1× coverage across 99.99% of the genome and ≥10× coverage across 90.10% of genomic positions. Regions exhibiting comparatively lower HiFi coverage primarily corresponded to portions of the control region (D-loop) and other AT-rich segments, which are commonly associated with reduced sequencing depth in mitochondrial genome assemblies. Nevertheless, no assembly discontinuities, unresolved gaps, or conflicting structural signals were detected, supporting the continuity and integrity of the reconstructed circular molecule.

External validation using independent Illumina short-read data (NCBI SRA: SRR30553176) obtained from a geographically distinct *H. plumierii* specimen further supported the structural consistency of the assembly. A total of 125,713 (0.058%)Illumina reads aligned successfully, achieving 99.99% genome coverage and a mean depth of 593.7×. Although these Illumina reads were not used for consensus generation due to potential population-level polymorphisms, the high mapping concordance and extensive genome coverage indicate strong overall sequence similarity between datasets and support the representative accuracy of the final mitochondrial genome assembly.

Structural annotation consistency was supported by MITOS2, which identified all 37 canonical mitochondrial genes with high Hidden Markov Model (HMM) scores. Ribosomal RNA and protein-coding genes (PCGs) exhibited extremely low e-values, confirming highly significant matches. While tRNA genes showed relatively higher e-values -expected given their short length and intrinsic structural variability- no patterns indicative of systematic mis-annotation or assembly artifacts were detected (Supplementary Figures S1 and S2). Finally, Tandem Repeat Finder detected only 82 bp of repetitive sequences across the complete mitochondrial genome, representing less than 0.5% of the assembled mitogenome. No tandem repeat arrays were detected within the mitochondrial control region (D-loop) under the parameters employed.

### 3.4. Phylogenetic Analysis and Comparative Sequence Similarity

**Figure 3:**
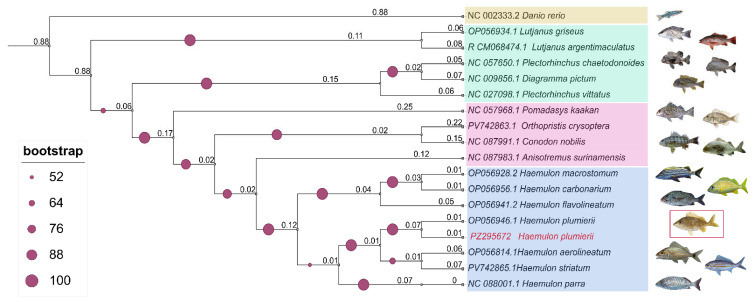
Maximum-likelihood phylogeny based on complete mitochondrial genomes. The phylogenetic tree was inferred using the maximum-likelihood method based on complete mitochondrial genome sequences. Numbers at nodes indicate bootstrap support values (1000 replicates). Colored boxes indicate the principal phylogenetic groups recovered in the analysis, including the *Haemulon* clade (blue), intermediate haemulid taxa (pink), external perciform taxa (green), and the outgroup *D. rerio* (beige).

Phylogenetic reconstruction based on complete mitochondrial genome sequences recovered a well-resolved topology consistent with the current systematics of the family *Haemulidae* (Figure 3). The newly assembled *H. plumierii* mitogenome clustered within the *Haemulon* clade and grouped closely with previously published *H. plumierii* sequences and other Caribbean haemulids, including *H. carbonarium* (OP056956.1), *H. flavolineatum* (OP056941.2), *H. macrostomum* (OP056928.2), *H. aurolineatum* (OP056814.1), *H. striatum* (PV742865.1), and *H. parra* (NC088001.1). Internal nodes within the *Haemulon* clade were generally supported by moderate to high bootstrap values, indicating a strong phylogenetic signal across the mitochondrial genome dataset.

The *Haemulon* clade was clearly separated from other haemulid genera, including *Anisotremus*, *Conodon*, *Orthopristis*, *Pomadasys*, and *Plectorhinchus*, whereas *D. rerio* formed a highly divergent outgroup lineage. The recovered topology was concordant with previously published phylogenetic reconstructions based on mitochondrial and multilocus datasets and supported the taxonomic placement of the newly sequenced *H. plumierii*. These phylogenetic relationships additionally reinforced the structural accuracy and evolutionary consistency of the assembled mitochondrial genome.

To complement phylogenetic inference, comparative BLAST analyses revealed high levels of overall sequence similarity among *Haemulon* mitogenomes, particularly across coding regions and ribosomal RNA genes. The newly assembled *H. plumierii* mitogenome showed strong similarity and near-complete alignment coverage relative to previously published mitochondrial genomes from the genus, supporting high conservation of mitochondrial architecture among Caribbean haemulids.

Based on phylogenetic proximity and mitochondrial genome similarity, a subset of seven closely related *Haemulon* species was selected for downstream comparative analyses, including mitochondrial synteny, codon usage bias, nucleotide diversity, and selective pressure assessments. Restricting these analyses to closely related taxa minimizes phylogenetic noise and enables a finer-scale evaluation of mitochondrial evolutionary patterns within the genus.

**Figure 4:**
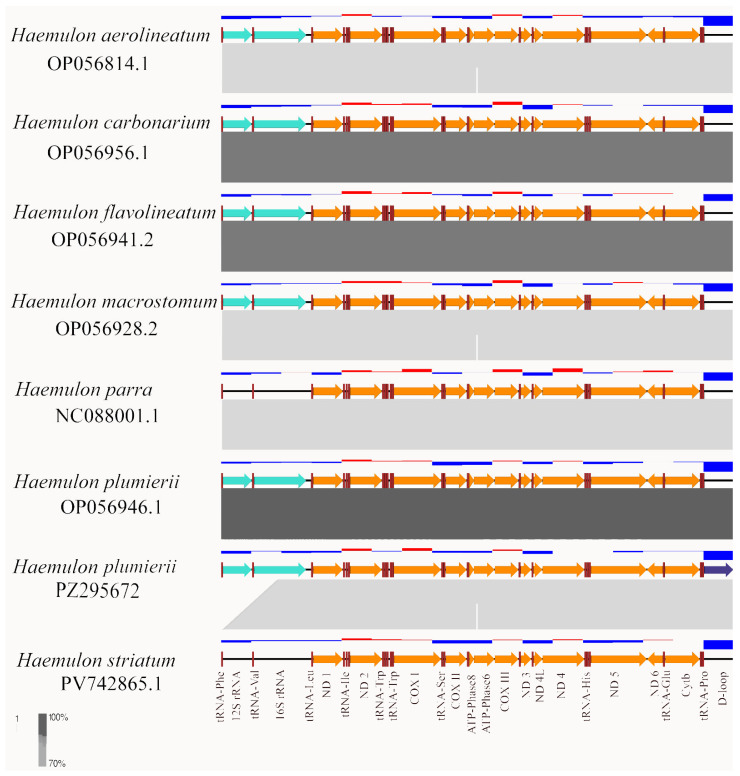
Structural comparison of mitochondrial genomes from closely related Caribbean species of *Haemulon*. Protein-coding genes, rRNAs, and tRNAs are shown as arrows indicating gene position and transcriptional orientation. Shaded blocks between genomes represent BLASTn alignments generated in Easyfig (minimum alignment length = 100 bp, minimum identity = 70%, maximum E-value = 1 × 10^−5^), with darker shading indicating higher nucleotide sequence identity (70–100%). The upper panel displays GC content across the mitogenome, calculated using a sliding window of 1000 bp with a step size of 200 bp. The purple arrow marks the control region (D-loop) in *H. plumierii* (PZ295672).

Comparative analyses of mitochondrial gene order among the selected *Haemulon* species revealed a high degree of synteny and structural conservation across mitogenomes (Figure 4). All analyzed taxa exhibited the canonical vertebrate mitochondrial gene complement and organization, including 13 protein-coding genes, 22 tRNA genes, and two ribosomal RNA genes, without evidence of large-scale rearrangements or gene inversions.

Linear synteny maps revealed strong conservation of gene boundaries, transcriptional orientation, and overall genomic organization across all compared taxa. Only minor variation was observed in portions of the ribosomal RNA regions and the control region (D-loop). The complete collinearity observed between the newly assembled *H. plumierii* mitogenome and previously published *Haemulon* mitogenomes further supported the quality and structural consistency of the assembly and annotation processes.

### 3.5. Selection Pressure Analysis of Mitochondrial Protein-Coding Genes

Selective pressures acting on mitochondrial protein-coding genes were assessed using nonsynonymous (dN), synonymous (dS), and dN/dS (ω) substitution rates across seven closely related *Haemulon* species selected based on phylogenetic proximity and mitochondrial genome similarity. To facilitate functional interpretation, results were summarized according to oxidative phosphorylation (OXPHOS) complexes, including Complex I (NADH dehydrogenase), Complex III (cytochrome b), Complex IV (cytochrome c oxidase), and Complex V (ATP synthase) (Table 4).

Across all mitochondrial complexes, ω values were consistently well below 1, indicating pervasive purifying selection acting on mitochondrial genes. This pattern is characteristic of vertebrate mitogenomes and reflects the essential role of mitochondrial proteins in oxidative phosphorylation, cellular respiration, and organismal energy metabolism. Among complexes, the lowest mean ω values were observed in Complex IV (COX genes), followed by Complex III (COB), whereas comparatively higher—although still low—ω values were detected in Complex I (NADH genes) and Complex V (ATP synthase genes).

The corrected Complex I dataset, including nad1, nad2, nad3, nad4, nad4l, nad5, and nad6, exhibited a mean dN/dS ratio of 0.083 ± 0.117. Within this complex, nad5 and nad6 showed the highest ω values, indicating comparatively relaxed selective constraints relative to the highly conserved cytochrome oxidase genes. In contrast, Complex IV exhibited the strongest evolutionary conservation, consistent with the central role of cytochrome c oxidase in electron transfer and oxygen reduction during oxidative phosphorylation.

Despite these differences, the Kruskal–Wallis test did not detect significant differences in ω values among mitochondrial complexes (H = 4.19, *p* = 0.24), indicating that selective pressures remain broadly comparable across functional mitochondrial gene groups within the analyzed haemulid taxa. These results support long-term functional stability of mitochondrial OXPHOS pathways across Caribbean *Haemulon* lineages.

Amino acid sequence identity patterns further attested strong mitochondrial conservation. Most protein-coding genes exhibited very high mean amino acid identity values (≈100%) across the analyzed species, particularly genes associated with Complex III (cob), Complex IV (cox1–cox3), and several NADH subunits, including nad2, nad3, nad4, nad4l, and nad6. In contrast, atp6 (93.6%), nad1 (91.4%), and nad5 (90.5%) exhibited comparatively lower amino acid identity values, consistent with their relatively higher ω estimates. These genes have previously been identified among the more variable mitochondrial loci in teleost fishes and may experience relatively relaxed, although still predominantly purifying, selective constraints.

Overall, the combined dN/dS and amino acid identity analyses indicate strong functional conservation and pervasive purifying selection across mitochondrial OXPHOS genes in Caribbean haemulids. This pattern is consistent with the high structural conservation observed among *Haemulon* mitogenomes and with the essential metabolic roles of mitochondrial respiratory-chain proteins.

### 3.6. Codon Usage Bias, Nucleotide Diversity, and Genetic Distance Analyses

Patterns of codon usage in mitochondrial protein-coding genes were evaluated using relative synonymous codon usage (RSCU) values summarized across oxidative phosphorylation (OXPHOS) complexes to facilitate functional interpretation (Table 5). Across all analyzed haemulid mitogenomes, codon usage exhibited a clear and consistent bias toward A- and T-ending codons, reflecting the overall AT-rich composition characteristic of vertebrate mitochondrial genomes.

**Figure 5:**
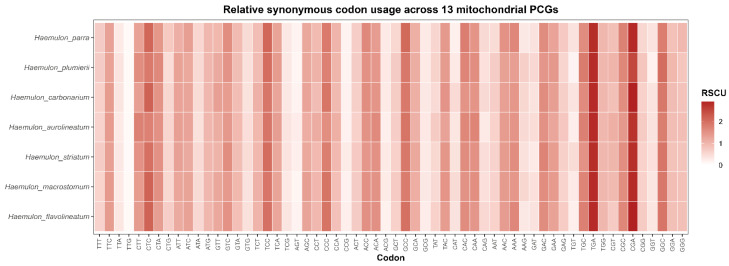
Heatmap of relative synonymous codon usage (RSCU) values across the 13 mitochondrial protein-coding genes of selected haemulid species. Warmer colors indicate higher codon usage frequencies. Stop codons were excluded from the analysis.

Heatmap visualization of RSCU values revealed highly similar codon usage profiles among *Haemulon* species, supporting strong conservation of mitochondrial coding patterns within the genus (Figure 5). Codons associated with leucine, serine, threonine, alanine, and glycine frequently exhibited elevated RSCU values across taxa. This AT-driven codon usage bias was particularly pronounced in Complex I (NADH dehydrogenase) and Complex V (ATP synthase), which comprise the largest number of mitochondrial genes and display preferential use of synonymous codons ending in A or T.

In contrast, genes belonging to Complex IV (cox1–cox3) exhibited comparatively constrained codon usage profiles, with fewer codons showing strong overrepresentation (RSCU > 1.5). Complex III (cob) displayed intermediate codon usage patterns, combining strong AT preference with lower overall RSCU variability relative to Complex I genes. Overall, codon usage variation among mitochondrial complexes paralleled patterns of sequence conservation and selective constraint inferred from dN/dS analyses.

**Figure 6:**
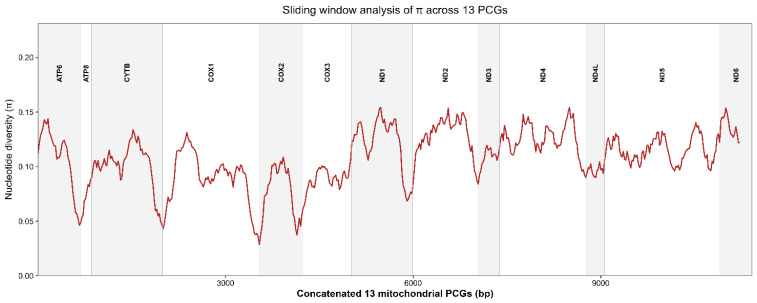
Sliding-window analysis of nucleotide diversity (π) across concatenated mitochondrial protein-coding genes from selected haemulid species. Analyses were performed using a 200 bp window size and a 20 bp step size.

Sliding-window analyses of nucleotide diversity across the concatenated alignment of the 13 mitochondrial protein-coding genes revealed heterogeneous patterns of sequence variability along the mitogenome (Figure 6). Nucleotide diversity (π) values ranged from approximately 0.028 to 0.154 across the analyzed regions, indicating overall moderate mitochondrial divergence among haemulid taxa.

The most conserved regions corresponded primarily to cytochrome c oxidase genes (cox1–cox3), whereas elevated nucleotide diversity was observed in several NADH dehydrogenase genes, particularly nad5 and portions of nad1 and nad6. These patterns were consistent with the selective pressure analyses, which identified lower ω values and higher amino acid conservation in COX genes relative to NADH subunits.

**Figure 7:**
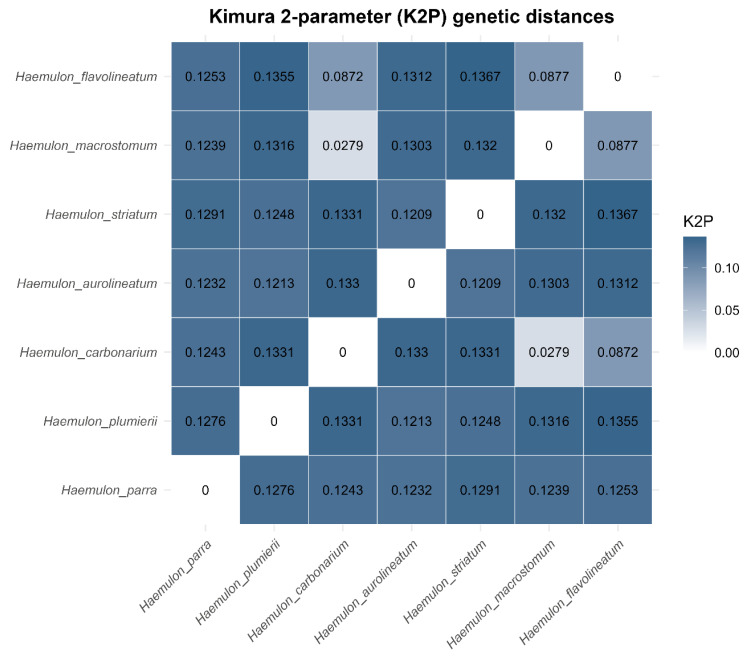
Heatmap of pairwise Kimura 2-parameter (K2P) genetic distances among selected haemulid mitochondrial genomes based on concatenated mitochondrial protein-coding gene sequences.

Pairwise genetic distance analyses based on the Kimura 2-parameter (K2P) model further supported moderate interspecific divergence among *Haemulon* species and related haemulid taxa (Figure 7). Lower K2P distances were observed among closely related Caribbean *Haemulon* species, whereas higher divergence values characterized comparisons involving more distantly related haemulid genera and the outgroup *D. rerio*.

Overall, the combined RSCU, nucleotide diversity, and K2P analyses indicate that haemulid mitochondrial genomes are characterized by strong compositional conservation, pervasive purifying selection, and moderate lineage-specific divergence patterns associated with mitochondrial evolution.

### 3.7. D-Loop Characterization

The mitochondrial control region (D-loop) was identified as the largest non-coding intergenic region within the analyzed mitogenomes and exhibited variation in both length and nucleotide composition among taxa (Table 6). In *H. plumierii*, the D-loop spanned 954 bp and was located between positions 15,870 and 16,823 of the mitochondrial genome. This length was consistent with those reported for other *Haemulon* species and with the previously published *H. plumierii* mitogenome, supporting structural conservation of the control region within the genus.

Across the analyzed haemulid taxa, D-loop length ranged from 840 bp in *Diagramma picta* to 1139 bp in *O. chrysoptera*. Species within the genus *Haemulon* exhibited a relatively narrow size range, varying from 947 bp in *H. aurolineatum* to 959 bp in *H. striatum*, whereas broader variation was observed among non-*Haemulon* haemulids.

Tandem repeat analyses identified only 82 bp of repetitive sequences in the *H. plumierii* mitogenome, representing less than 0.5% of the complete mitochondrial genome. The absence of extended repetitive regions supports the relatively conserved structural organization of the control region and suggests that D-loop variability among *Haemulon* species is primarily associated with nucleotide substitutions and minor length differences rather than expansion of large tandem repeat arrays.

The nucleotide composition of the *H. plumierii* D-loop was strongly AT-biased, with an overall A+T content of 61.01%. Similar AT enrichment patterns were consistently observed across haemulid mitogenomes, with A+T contents ranging from 57.22% in *C. nobilis* to 65.57% in *P. kaakan*. The outgroup *D. rerio* exhibited the highest A+T content (67.89%).

Analyses of nucleotide skews further revealed compositional asymmetry within the D-loop region (Table 6). In *H. plumierii*, the AT skew was slightly positive (0.007), whereas the GC skew was strongly negative (−0.199). Similar skew patterns were consistently observed among haemulid species, suggesting conserved strand-specific mutational and replication-associated biases within the mitochondrial control region.

## 4. Discussion

The complete mitochondrial genome assembled for *H. plumierii* exhibited the canonical vertebrate mitochondrial organization typical of haemulid mitogenomes [9,11]. The use of PacBio HiFi long-read sequencing enabled the recovery of a complete circular mitochondrial genome with high sequence continuity and consistent coverage across coding and non-coding regions, including the control region (D-loop), which is often difficult to reconstruct using short-read sequencing technologies alone [28,30]. Independent validation analyses, including read remapping, circularization checks, BLAST similarity analyses, and comparative synteny, further supported the accuracy and completeness of the assembly. The low abundance of tandem repeats detected in the mitogenome likely contributed to the successful reconstruction of the control region and supports the relatively stable organization observed within *Haemulon* mitogenomes.

Comparative analyses revealed highly conserved mitochondrial gene order, transcriptional orientation, and nucleotide composition across the analyzed haemulid taxa [12,56,57]. All examined *Haemulon* species exhibited the typical vertebrate complement of 13 protein-coding genes, 22 tRNAs, and two ribosomal RNA genes without evidence of large-scale rearrangements or gene losses [9,11]. Additionally, *Haemulon* species displayed comparable A+T contents and highly similar AT and GC skew values, suggesting conserved strand-specific mutational pressures and replication-associated compositional biases within the genus [9,58]. The consistently negative GC skew values observed across haemulid mitogenomes likely reflect the asymmetric replication mechanism characteristic of vertebrate mitochondrial DNA, in which prolonged exposure of the heavy strand in a single-stranded state promotes spontaneous cytosine and adenine deamination, contributing to nucleotide compositional asymmetry [9,11].

Notably, several protein-coding genes terminated with incomplete stop codons (T or TA), a common feature of vertebrate mitochondrial genomes. According to the “punctuation model” of mitochondrial RNA processing, these truncated codons are converted into functional TAA stop codons through post-transcriptional polyadenylation [59]. Likewise, although most mitochondrial tRNAs exhibited the canonical cloverleaf secondary structure, tRNA-Ser(AGN) lacked a complete DHU arm, a structural feature widely reported in vertebrate mitogenomes that is not thought to compromise mitochondrial translation due to compensatory interactions within the mitoribosome [60].

Phylogenetic analyses based on complete mitochondrial genomes recovered a topology consistent with the current systematics of *Haemulidae* and supported the placement of *H. plumierii* within the Caribbean *Haemulon* clade. The close clustering of *H. plumierii* with *H. parra*, *H. carbonarium*, *H. flavolineatum*, *H. macrostomum*, *H. striatum*, and *H. aurolineatum* agrees with previous phylogenetic studies based on mitochondrial and multilocus datasets and reinforces the evolutionary coherence of Caribbean haemulids [61,62]. The high degree of mitochondrial synteny and overall sequence similarity observed among these taxa further supports the strong conservation of mitochondrial genome organization within the genus.

Analyses of selective pressures revealed pervasive purifying selection acting across all mitochondrial oxidative phosphorylation complexes, as indicated by ω (dN/dS) values consistently below 1. Complex IV genes (*cox1*–*cox3*) exhibited the lowest ω values and the highest amino acid conservation, consistent with the central role of cytochrome c oxidase in electron transport and aerobic metabolism [11,63]. In contrast, several NADH dehydrogenase genes, particularly *nad5* and *nad6*, exhibited comparatively higher ω values and elevated nucleotide diversity, although they remained under strong purifying selection overall [3]. Similar evolutionary patterns have been widely reported in teleost mitogenomes, where NADH-associated genes generally evolve more rapidly than cytochrome oxidase genes while maintaining strong metabolic constraints [11].

Patterns of codon usage further supported the influence of nucleotide compositional bias on haemulid mitochondrial genome evolution. Relative synonymous codon usage (RSCU) analyses revealed a clear preference for A- and T-ending codons across mitochondrial protein-coding genes, particularly within Complex I and Complex V. Similar codon usage patterns have been widely documented in teleost mitochondrial genomes and are generally attributed to mutational pressure associated with strand-specific nucleotide composition rather than translational optimization [9,64]. In contrast, cytochrome oxidase genes displayed comparatively constrained codon usage profiles, consistent with their low dN/dS ratios and high amino acid conservation [63].

Sliding-window analyses of nucleotide diversity identified heterogeneous patterns of sequence divergence across the mitochondrial genome. Regions corresponding to *cox1*–*cox3* displayed the lowest nucleotide diversity values, reinforcing their utility as highly conserved mitochondrial markers. Conversely, elevated variability was observed in portions of *nad5*, *nad1*, and *nad6*, suggesting that these genes may provide greater phylogenetic resolution for closely related taxa and population-level studies [65]. Pairwise K2P genetic distances similarly revealed moderate interspecific divergence among Caribbean *Haemulon* species while maintaining clear separation from more distantly related haemulid genera and the outgroup *D. rerio*. Together, these patterns indicate that different mitochondrial regions evolve under distinct levels of evolutionary constraint and may therefore be useful for complementary taxonomic and evolutionary applications [11,66].

The mitochondrial control region (D-loop) exhibited comparatively higher A+T content relative to the complete mitogenome and showed conserved compositional characteristics among *Haemulon* species. Although moderate variation in D-loop length was observed among haemulid taxa, species within the genus *Haemulon* displayed relatively narrow size ranges and highly similar nucleotide composition profiles. Tandem Repeat Finder analyses detected no tandem repeat arrays within the D-loop region of *H. plumierii*, suggesting that control-region variation in these species is driven primarily by nucleotide substitutions and small indels rather than major repetitive expansions [9,11]. Similar patterns have been reported in other teleost mitochondrial genomes and may reflect conserved structural organization of the mitochondrial control region [67,68].

Since *H. plumierii* has previously been proposed as a bioindicator species within the Mesoamerican Reef System Synoptic Monitoring Program (SAM), the mitogenomic resource presented here provides an important genomic framework for future ecological, evolutionary, and environmental genomics studies [69]. Although the mitochondrial genome itself cannot be proposed as a direct biomarker of environmental stress, it establishes the necessary genomic foundation for future transcriptomic, population-genomic, phylogeographic, and mitochondrial physiology studies evaluating contaminant responses and environmental stress in reef-associated fishes. Given the central role of mitochondrial genes in cellular respiration and oxidative phosphorylation, the complete mitogenome of *H. plumierii* may facilitate future investigations exploring mitochondrial dysfunction, oxidative stress responses, and contaminant exposure in Caribbean reef ecosystems [14,15].

## 5. Conclusions

This study presents a high-quality complete mitochondrial genome for *H. plumierii* generated using PacBio HiFi long-read sequencing, providing a robust genomic resource for comparative and evolutionary analyses within *Haemulidae*. The assembled mitogenome exhibited strong structural conservation, stable nucleotide composition, conserved mitochondrial synteny, and pervasive purifying selection across oxidative phosphorylation genes. Comparative analyses additionally revealed highly conserved amino acid sequences, codon usage patterns, and mitochondrial genome organization among closely related Caribbean *Haemulon* species.

The combined analyses of selective pressures, codon usage bias, nucleotide diversity, and genetic divergence indicate that haemulid mitochondrial genomes are shaped primarily by strong functional constraints associated with mitochondrial respiration and energy metabolism. At the same time, moderately variable regions identified in several NADH dehydrogenase genes may provide useful molecular markers for future phylogenetic, phylogeographic, and population-level studies within the genus.

Given its ecological importance, broad Caribbean distribution, and previous designation as a bioindicator species within the SAM, *H. plumierii* represents a valuable model for future environmental monitoring applications. Although the mitochondrial genome itself is not proposed as a direct biomarker of environmental stress, the mitogenomic resource generated here establishes an essential evolutionary and functional framework for future studies investigating mitochondrial responses to contaminants, environmental perturbations, and reef ecosystem health.

## Figures and Tables

**Table 1:** Functional classification and genomic features of mitochondrial genes in *H. plumierii*.

Gene	Start	Stop	Start Codon	Stop Codon	Strand	Size (bp)	Function
*nd1*	3032	4006	ATG	TAA	+	975	NADH oxidation and proton pumping
*nd2*	4245	5290	ATG	T (incomplete)	+	1046	NADH oxidation and proton pumping
*cox1*	5683	7233	GTG	AGG	+	1551	Terminal electron transfer to O_2_
*cox2*	7383	8073	ATG	T (incomplete)	+	691	Terminal electron transfer to O_2_
*atp8*	8150	8317	ATG	TAA	+	168	ATP synthesis
*atp6*	8308	8990	ATG	T (incomplete)	+	683	ATP synthesis
*cox3*	8991	9775	ATG	T (incomplete)	+	785	Terminal electron transfer to O_2_
*nd3*	9848	10,196	ATG	T (incomplete)	+	349	NADH oxidation and proton pumping
*nd4l*	10,266	10,562	ATG	TAA	+	297	NADH oxidation and proton pumping
*nd4*	10,556	11,936	ATG	T (incomplete)	+	1381	NADH oxidation and proton pumping
*nd5*	12,151	13,989	ATG	TAA	+	1839	NADH oxidation and proton pumping
*nd6*	13,986	14,507	ATG	AGG	−	522	NADH oxidation and proton pumping
*cytb*	14,584	15,724	ATG	T (incomplete)	+	1141	Electron transfer (bc_1_ complex)
*rrns (12s rRNA)*	70	1019			+	950	Mitochondrial translation
*rrnl (16s rRNA)*	1092	2799			+	1708	Mitochondrial translation
*control region (D-loop)*	15,870	16,822			+	953	Replication and transcription regulation

(+) indicates heavy (H) strand, (−) indicates light (L) strand.

**Table 2:** Genomic features of mitochondrial transfer RNA (tRNA) genes in *H. plumierii*.

tRNA	Start	Stop	Strand	Size (bp)	Anticodon
*tRNA-Phe*	1	69	+	69	GAA
*tRNA-Val*	1020	1091	+	72	TAC
*tRNA-Leu (UUR)*	2958	3031	+	74	TAA
*tRNA-Ile*	4008	4077	+	70	GAT
*tRNA-Gln*	4095	4165	−	71	TTG
*tRNA-Met*	4176	4244	+	69	CAT
*tRNA-Trp*	5291	5362	+	72	TCA
*tRNA-Ala*	5364	5432	−	69	TGC
*tRNA-Asn*	5434	5506	−	73	GTT
*tRNA-Cys*	5544	5610	−	67	GCA
*tRNA-Tyr*	5611	5681	−	71	GTA
*tRNA-Ser (UCN)*	7234	7304	−	71	TGA
*tRNA-Asp*	7308	7378	+	71	GTC
*tRNA-Lys*	8074	8148	+	75	TTT
*tRNA-Gly*	9776	9847	+	72	TCC
*tRNA-Arg*	10,197	10,265	+	69	TCG
*tRNA-His*	11,937	12,005	+	69	GTG
*tRNA-Ser (AGY)*	12,006	12,073	+	68	GCT
*tRNA-Leu (CUN)*	12,078	12,150	+	73	TAG
*tRNA-Glu*	14,508	14,577	−	70	TTC
*tRNA-Thr*	15,725	15,796	+	72	TGT
*tRNA-Pro*	15,796	15,869	−	74	TGG

**Table 3:** Genome size, nucleotide composition and compositional skews of mitochondrial genomes from this study.

Species	Accession No.	Length (bp)	GC%	A (%)	T (%)	G (%)	C (%)	A+T (%)	G+C (%)	AT Skew	GC Skew
*H. plumierii*	PZ295672	16,823	48.78	26.04	25.19	17.55	31.23	51.22	48.78	0.017	−0.280
*Haemulon aurolineatum*	OP056814.1	16,810	49.57	25.95	25.42	17.68	30.95	51.37	48.63	0.01	−0.273
*Haemulon macrostomum*	OP056928.2	16,818	49.16	25.94	24.9	17.59	31.57	50.84	49.16	0.02	−0.284
*Haemulon flavolineatum*	OP056941.2	16,822	49.48	25.66	24.86	17.83	31.65	50.52	49.48	0.016	−0.279
*Haemulon carbonarium*	OP056956.1	16,819	49.16	25.85	24.98	17.72	31.44	50.84	49.16	0.017	−0.279
*Haemulon parra*	NC088001.1	16,820	49.57	25.86	24.57	17.66	31.91	50.43	49.57	0.025	−0.288
*H. plumierii*	OP056946.1	16,823	48.72	26.05	25.23	17.57	31.15	51.28	48.72	0.016	−0.279
*Haemulon striatum*	PV742865.1	16,823	48.91	25.99	25.1	17.68	31.23	51.09	48.91	0.018	−0.277
*O. chrysoptera*	PV742863.1	16,985	47.74	26.62	25.63	17.42	30.32	52.26	47.74	0.019	−0.270
*A. surinamensis*	NC_087983.1	16,845	48.05	27.11	24.84	16.55	31.5	51.95	48.05	0.044	−0.311
*P. kaakan*	NC_057968.1	16,808	48.2	27.12	24.67	16.55	31.65	51.8	48.2	0.047	−0.313
*Plectorhinchus chaetodonoides*	NC_057650.1	16,546	47.49	28.06	24.46	16.28	31.21	52.51	47.49	0.068	−0.314
*Plectorhinchus vittatus*	NC_027098.1	16,545	47.31	27.98	24.71	16.38	30.93	52.69	47.31	0.062	−0.308
*C. nobilis*	NC_087991.1	16,956	49.81	26.17	24.02	17.56	32.25	50.19	49.81	0.043	−0.295
*Diagramma pictum*	NC_009856.1	16,530	47.81	27.55	24.64	16.88	30.93	52.19	47.81	0.056	−0.294
*Lutjanus griseus*	OP056934.1	16,538	46.92	28.24	24.84	16.14	30.78	53.08	46.92	0.064	−0.312
*L. argentimaculatus*	CM068474.1	16,634	39.5	24.5	28.08	31.19	16.23	52.59	47.41	−0.068	0.316
*D. rerio*	NC_002333.2	16,596	39.93	31.94	28.13	16.02	23.92	60.07	39.93	0.063	−0.198

**Table 4:** Estimates of dN, dS, and dN/dS (ω) for mitochondrial protein-coding genes grouped by OXPHOS complexes.

Complex	N Genes	dN (Mean ± SD)	dS (Mean ± SD)	dN/dS (Mean ± SD)
I (NADH)	7	0.024 ± 0.026	0.318 ± 0.256	0.083 ± 0.117
III (COB)	1	0.008 ± 0.00	0.342 ± 0.000	0.024 ± 0.00
IV (COX)	3	0.00010 ± 0.00	0.083 ± 0.025	0.001 ± 0.00
V (ATP)	2	0.023 ± 0.023	1.819 ± 2.414	0.007 ± 0.008

**Table 5:** Relative synonymous codon usage patterns in mitochondrial protein-coding genes grouped by mitochondrial complex.

Mitochondrial Complex	Genes	Mean RSCU ± SD	Codons with RSCU > 1.5
Complex I (NADH)	*nad1*–*nad6*	1.06 ± 0.60	ACC, CAA, CCC, CGA, CGC, CTC, GCC, GGC, TCC, TGA, TGC
Complex III (COB)	*cob*	1.07 ± 0.81	AAA, ACA, ATC, CAC, CCC, CGA, CGC, CTA, CTC, GAC, GCC, GGC, GTC, TAC, TCC, TGA, TGC, TTC
Complex IV (COX)	*cox1*–*cox3*	1.07 ± 0.65	AAA, ACA, CAA, CAC, CCC, CGA, CGC, CGT, CTA, CTC, CTT, GAC, GCC, GGC, TCA, TCC, TGA, TGC
Complex V (ATP)	*atp6*–*atp8*	1.06 ± 0.84	AAA, ACC, AGC, CAC, CGA, CTA, CTC, GAC, GCC, GGC, GTC, TAC, TCC, TGA

**Table 6:** Genomic position, length, and nucleotide composition of the mitochondrial control region (D-loop) across analyzed species.

Species	D-Loop Start	D-Loop End	D-Loop Length bp	A%	T%	G%	C%	A+T%	G+C%	AT Skew	GC Skew
*H. plumierii* *PZ295672*	15,870	16,823	954	30.71	30.29	15.62	23.38	61.01	38.99	0.007	−0.199
*Haemulon* *Parra*	15,866	16,820	955	30.26	31.2	16.23	22.3	61.47	38.53	−0.015	−0.158
*Lutjanus griseus*	15,672	16,538	867	32.99	30.57	15.8	20.65	63.55	36.45	0.038	−0.133
*P kaakan*	15,807	16,808	1002	33.63	31.94	12.67	21.76	65.57	34.43	0.026	−0.264
*O chrysoptera*	15,847	16,985	1139	31.17	30.9	16.59	21.33	62.07	37.93	0.004	−0.125
*Haemulon carbonarium*	15,865	16,819	955	30.37	29.63	17.59	22.41	60	40	0.012	−0.12
*P chaetodonoides*	15,699	16,546	848	30.42	27	15.45	27.12	57.43	42.57	0.06	−0.274
*Plectorhinchus vittatus*	15,697	16,545	849	29.56	29.09	15.43	25.91	58.66	41.34	0.008	−0.254
*Haemulon aurolineatum*	15,864	16,810	947	30.83	31.26	15.95	21.96	62.09	37.91	−0.007	−0.159
*D. rerio*	1	950	950	33.68	34.21	13.58	18.53	67.89	32.11	−0.008	−0.154
*C nobilis*	15,891	16,956	1066	27.95	29.27	15.95	26.83	57.22	42.78	−0.023	−0.254
*A surinamensis*	15,906	16,845	940	31.49	33.09	14.36	21.06	64.57	35.43	−0.025	−0.189
*H. plumierii OP056946.1*	15,870	16,823	954	30.82	30.92	15.51	22.75	61.74	38.26	−0.002	−0.189
*Diagramma picta*	15,691	16,530	840	31.31	29.76	14.4	24.52	61.07	38.93	0.025	−0.26
*Haemulon striatum*	15,865	16,823	959	30.87	31.07	16.16	21.9	61.94	38.06	−0.003	−0.151
*Haemulon macrostomum*	15,865	16,818	954	30.19	30.82	16.67	22.33	61.01	38.99	−0.01	−0.145
*Haemulon flavolineatum*	15,868	16,822	955	29.32	30.16	18.22	22.3	59.48	40.52	−0.014	−0.101

## Data Availability

The complete mitochondrial genome assembly and annotation of *H. plumierii* are available in GenBank under accession number PZ295672, associated with BioProject PRJNA1451883 and BioSample SAMN57202943. All the subsequent data can be available on request.

## Supplementary Materials

The following supporting information can be downloaded at: https://www.mdpi.com/article/10.3390/genes17050585/s1, Supplementary Table S1: Results of Blast analysis of complete mitogenomes respect to *H. plumierii*. Figure S1: Detailed mitochondrial genome annotation map of *H. plumierii* generated using MITOS on the Galaxy platform. The position and genomic extent of the 37 expected mitochondrial genetic elements are shown, including 13 protein-coding genes, 22 transfer RNA (tRNA) genes, and two ribosomal RNA (rRNA) genes. The x-axis represents genomic coordinates (bp), while the y-axis (logarithmic scale) indicates HMM alignment scores produced by MITOS. Continuous blocks with high scores correspond to well-supported annotations, whereas discontinuous blocks or lower scores may reflect regions with reduced coverage, partial sequences, or potential structural variability in tRNA genes. Symbols above the map indicate the genomic positions of tRNA genes. Figure S2 Distribution of e-values by genomic position for MITOS-based mitochondrial genome annotations. Labeled points correspond to individual genes, including tRNAs, rRNAs, and protein-coding genes (PCGs). The y-axis is shown on a logarithmic scale to facilitate visualization of annotation significance. No e-value patterns indicative of widespread false-positive annotations were detected.

## Abbreviations

The following abbreviations are used in this manuscript:

ATP	Adenosine triphosphate
bp	Base pairs
CDS	Coding sequence
D-loop	Displacement loop (control region)
dN	Nonsynonymous substitution rate
dS	Synonymous substitution rate
dN/dS (ω)	Ratio of nonsynonymous to synonymous substitutions
DNA	Deoxyribonucleic acid
GoM	Gulf-of-Mexico
GC	Guanine–cytosine content
HiFi	High-fidelity reads (PacBio)
H strand	Heavy strand
L strand	Light strand
MAFFT	Multiple Alignment using Fast Fourier Transform
MFannot	Mitochondrial genome annotation tool
MITOS	Mitochondrial genome annotation server
ML	Maximum likelihood
mtDNA	Mitochondrial DNA
NCBI	National Center for Biotechnology Information
OXPHOS	Oxidative phosphorylation
PacBio	Pacific Biosciences
PCG	Protein-coding gene
PBS	Phosphate-buffered saline
RSCU	Relative synonymous codon usage
rRNA	Ribosomal RNA
SNP	Single nucleotide polymorphism
tRNA	Transfer RNA
tRNAscan-SE	Transfer RNA Scan–Search Engine
WAF	Water-accommodated fraction
EAF	Emulsified aqueous fraction
