# Supplementary material for: Complete Mitochondrial Genome of Haemulon plumierii (Lacepède, 1801) Supports Its Use as a Sentinel Reef Fish
Source: Genes (Basel). 2026 May 20;17(5):585. doi: 10.3390/genes17050585 (PMC13206589; doi:10.3390/genes17050585)
Supplement: Supplementary file 1 [file genes-17-00585-s001.zip › genes-4299388-supplementary.pdf]

genes

MDPI

Supplementary Table S1. Results of Blast analysis of complete mitogenomes respect to *H. plumierii*

Query species	Subject species	Accession	Identity (%)	Alignment length (bp)	Mismatches	Gaps	E-value	Bit score
H. plumierii PZ295672	*Haemulon plumierii*	OP056946.1	**98.28**	**16,824**	288	2	0	29,460
H. plumierii PZ295672	*Haemulon aurolineatum*	OP056814.1	90.84	16,817	1,515	21	0	22,495
H. plumierii PZ295672	*Haemulon striatum*	PV742865.1	90.46	16,840	1,573	29	0	22,164
H. plumierii PZ295672	*Haemulon parra*	PP032976.1	90.4	16,821	1,588	25	0	22,090
H. plumierii PZ295672	*Haemulon macrostomum*	OP056928.2	89.87	16,842	1,664	39	0	21,605
H. plumierii PZ295672	*Haemulon flavolineatum*	OP056941.2	89.8	16,839	1,682	31	0	21,545
H. plumierii PZ295672	*Haemulon carbonarium*	OP056956.1	89.77	16,840	1,685	35	0	21,518
H. plumierii PZ295672	*Orthopristis chrysoptera*	PV742863.1	84.34	15,917	2,373	94	0	15,477
H. plumierii PZ295672	*Anisotremus surinamensis*	NC_087983.1	85.05	12,935	1,895	32	0	13,136
H. plumierii PZ295672	*Conodon nobilis*	NC_087991.1	84.43	12,946	1,945	45	0	12,672
H. plumierii PZ295672	*Pomadasys kaakan*	NC_057968.1	83.14	12,948	2,099	74	0	11,740
H. plumierii PZ295672	*Plectorhinchus chaetodonoides*	NC_057650.1	80.76	12,887	2,368	85	0	9,956
H. plumierii PZ295672	*Plectorhinchus vittatus*	NC_027098.1	80.66	12,887	2,372	94	0	9,875
H. plumierii PZ295672	*Diagramma picta*	NC_009856.1	80.6	12,886	2,388	86	0	9,843
H. plumierii PZ295672	*Lutjanus griseus*	OP056934.1	80.81	12,959	2,366	95	0	10,041

 https://doi.org/10.3390/genes17050585

H. plumierii PZ295672	*Lutjanus argentimaculatus*	CM068474.1	80.67	7,124	1,320	50	0	5,474
H. plumierii PZ295672	*Danio rerio*	NC_002333.2	74.99	6,926	1,588	129	0	3,062

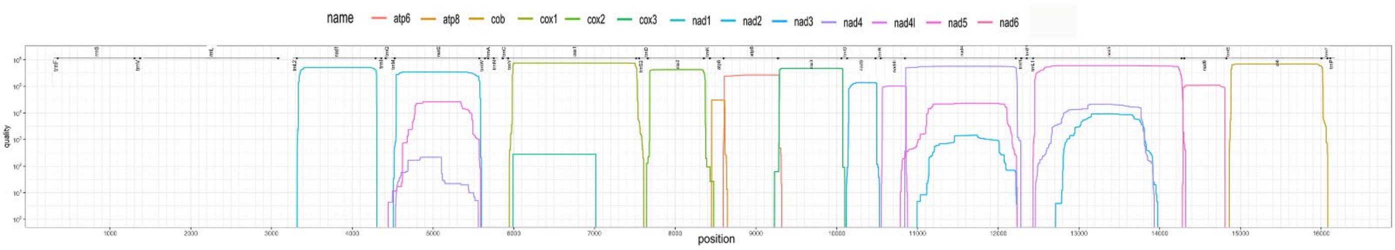

Figure S1. Detailed mitochondrial genome annotation map of *Haemulon plumierii* generated using MITOS on the Galaxy platform. The position and genomic extent of the 37 expected mitochondrial genetic elements are shown, including 13 protein-coding genes, 22 transfer RNA (tRNA) genes, and two ribosomal RNA (rRNA) genes. The x-axis represents genomic coordinates (bp), while the y-axis (logarithmic scale) indicates HMM alignment scores produced by MITOS. Continuous blocks with high scores correspond to well-supported annotations, whereas discontinuous blocks or lower scores may reflect regions with reduced coverage, partial sequences, or potential structural variability in tRNA genes. Symbols above the map indicate the genomic positions of tRNA genes.

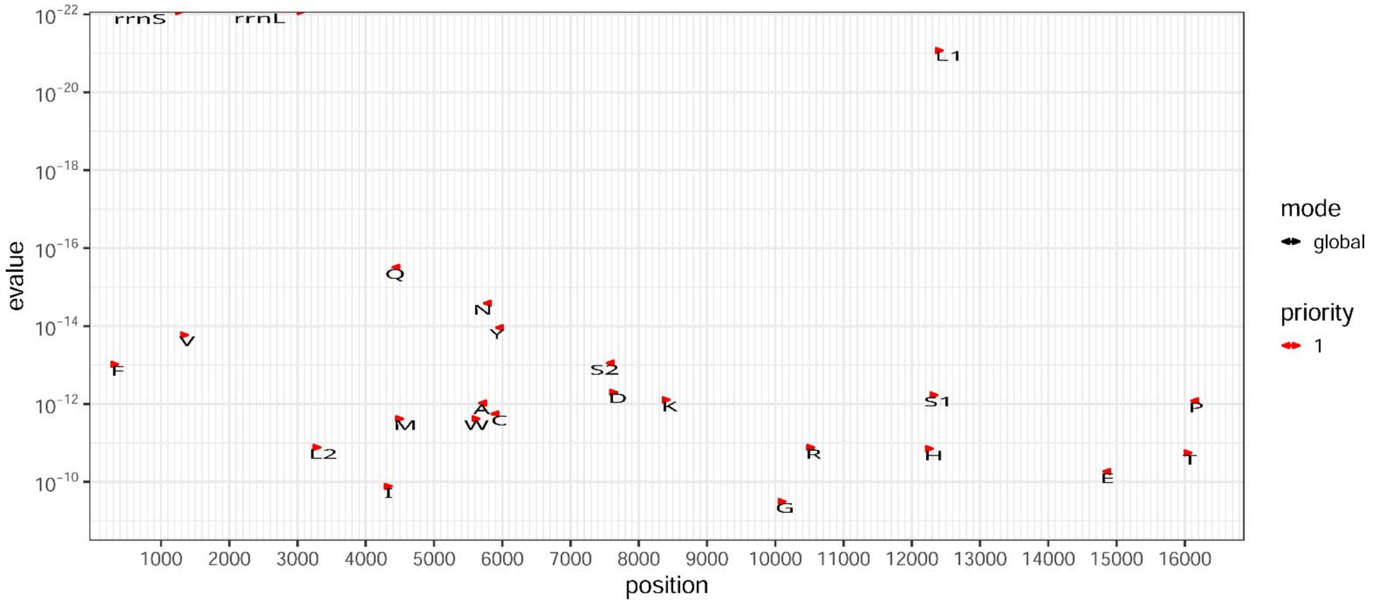

Figure S2. Distribution of e-values by genomic position for MITOS-based mitochondrial genome annotations. Labeled points correspond to individual genes, including tRNAs, rRNAs, and protein-coding genes (PCGs). The y-axis is shown on a logarithmic scale to facilitate visualization of annotation significance. No e-value patterns indicative of widespread false-positive annotations were detected.

https://doi.org/10.3390/genes17050585

Disclaimer/Publisher's Note: The statements, opinions and data contained in all publications are solely those of the individual author(s) and contributor(s) and not of MDPI and/or the editor(s). MDPI and/or the editor(s) disclaim responsibility for any injury to people or property resulting from any ideas, methods, instructions or products referred to in the content.

https://doi.org/10.3390/genes17050585
